# Differences in Durability of PARP Inhibition by Clinically Approved PARP Inhibitors: Implications for Combinations and Scheduling

**DOI:** 10.3390/cancers14225559

**Published:** 2022-11-12

**Authors:** Hannah L. Smith, Elaine Willmore, Asima Mukhopadhyay, Yvette Drew, Nicola J. Curtin

**Affiliations:** 1 Faculty of Medical Sciences, Newcastle University Centre for Cancer, Newcastle upon Tyne NE1 7RU, UK; 2 Kolkata Gynecological Oncology Trials and Translational Research Group, Chittaranjan National Cancer Institute, 37 SP Mukherjee Road, Kolkata 700026, India; 3 BC Cancer Centre Vancouver and Faculty of Medicine, University of British Columbia, Vancouver, BC V6T 1Z4, Canada

**Keywords:** PARP1, durability, DDR, ATR

## Abstract

**Simple Summary:**

PARPi are approved as monotherapy agents and under advanced clinical investigation as a combination therapy for the treatment of cancer. We demonstrate for the first time that five of the approved PARPi vary in their ability to suppress cellular PARP activity after drug removal. Rucaparib caused the most durable PARP inhibition, and olaparib and niraparib the least. Rucaparib enhanced ATR inhibitor cytotoxicity in sequential and co-exposures, whereas olaparib and niraparib were only active in co-exposure settings. These data have implications for the scheduling of PARPi clinically, particularly in combination with other drugs.

**Abstract:**

Six PARP inhibitors (PARPi) are approved for cancer therapy as monotherapy agents in daily or twice-daily continuous dosing schedules to maintain the necessary continuous suppression of PARP activity. Continuous PARP inhibition is required for single-agent anticancer activity. To investigate if such intense schedules are necessary, we determined the durability of PARP inhibition up to 72 h after a 1 h pulse of 1 µM of five of the approved PARPi, rucaparib, olaparib, niraparib, talazoparib and pamiparib, in IGROV-1 and ES-2 (human ovarian cancer) cells. Rucaparib caused the most persistent inhibition of PARP activity when maintained at ≥75% at 72 h after drug withdrawal in both IGROV-1 and ES-2 cells, but inhibition was more rapidly lost with the other PARPi. PARPi are also under clinical investigation with ATR inhibitors, and thus, we evaluated the implications for scheduling with an ATR inhibitor (VE-821). Rucaparib enhanced VE-821 cytotoxicity in co-exposure, sequential and delayed (24 h drug-free) schedules in IGROV-1 and ES-2 cells. Olaparib and niraparib enhanced VE-821 cytotoxicity only in co-exposed cells and not in sequential exposures. These data have clinical implications for the scheduling of PARPi as a monotherapy and in combination with ATR inhibitors and other cytotoxic drugs.

## 1. Introduction

**Figure 1:**
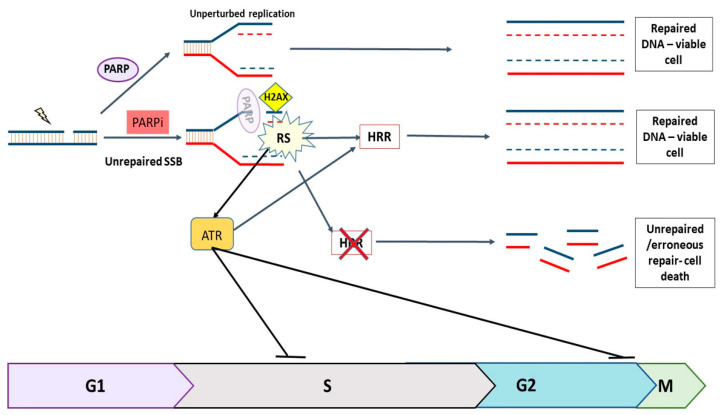
The role of PARP1 and PARP2 (PARP) and ATR in the DNA damage response. Endogenously generated SSBs are continuously repaired by PARP-dependent repair mechanisms. When PARP is inhibited, unrepaired SSBs collide with replication forks, causing them to stall and collapse, resulting in DSBs which can only be repaired by HRR during S and early G2 phases. If HRR is defective, e.g., due to a *BRCA* mutation, the DNA cannot be repaired accurately, resulting in cell death. Replication stress (RS) caused by PARPi activates ATR, which triggers a cascade that halts cell cycle progression and promotes HRR.

PARP inhibitors (PARPi) are a class of anticancer drug that work by both inhibiting the repair of DNA single-strand breaks (SSBs) and trapping PARP1 and PARP2 at the site of the break [1,2]. This results in replication-associated lesions that both activate ATR and the DNA damage cell cycle checkpoint cascade, and which are resolved by homologous recombination DNA repair (HRR: Figure 1) [3,4,5].

PARPi exploit tumour-specific defects in HRR, e.g., *BRCA* mutations, by a process known as synthetic lethality [6,7,8]. The first approvals for PARPi by the FDA started in 2014 with olaparib, followed by rucaparib, niraparib and talazoparib (https://www.fda.gov, accessed on 28 September 2022) and subsequent approvals by the European Medicines Agency. The PARPi pamiparib and fluzoparib have been approved in China [9,10]. They are approved as single agents for cancers associated with defective HRR (HRD): ovarian, breast, castrate-resistant prostate and pancreatic cancer. These and other PARPi are in advanced clinical trials, and numerous trials are investigating combinations with cytotoxic and molecularly targeted therapy, including ATR inhibitors (https://clinicaltrials.gov, accessed on 28 September 2022). All approved PARPi are currently given continuously on a once daily or twice daily schedule because, for effective single-agent activity, PARP must be completely and continuously inhibited so that cells cannot repair DNA breaks before S-phase progression [11,12,13]. However, continuous inhibition may not be appropriate for combinations with genotoxic agents. It is becoming apparent that differences between the PARPi exist, in terms of their specificity, potency and “trapping” ability, which may underlie some of the differences in their activity and toxicity clinically [14,15,16,17]. To date, no study has investigated the potential differences in the durability of PARP inhibition between the approved PARPi. Our previous studies indicated that rucaparib induced durable PARP inhibition in patients [18] and that a weekly schedule was as effective as daily dosing against *BRCA2* mutant xenografts [19].

The purpose of the work reported here was to determine if persistent PARP inhibition was a class effect or unique to rucaparib using a GCLP-validated cell-based PARP activity assay. We used the clinically approved PARPi: olaparib, rucaparib, niraparib, talazoparib and pamiparib in IGROV-1 cells; and rucaparib, olaparib and niraparib in ovarian ES-2 cells. These two well-characterised ovarian cancer cell lines of differing molecular pathologies [20,21] were selected because four of the five PARPi used in this study are approved in ovarian cancer. They are both considered to be wildtype for *BRCA1* and *BRCA2* [22] and are HRR proficient and resistant to rucaparib-induced cytotoxicity [23]. They are not reported to overexpress multidrug-resistance transporters.

PARPi and ATR inhibitor combinations have been shown to be synergistic preclinically and are being investigated in several clinical trials. The impact of scheduling on the synergy with ATR inhibitors was, therefore, also investigated. We discovered that rucaparib was unique in its ability to cause sustained high levels of PARP inhibition and was effective in sequential administration with the ATR inhibitor, VE-821. These data have clinical implications for the scheduling of PARPi as a monotherapy and in combination.

## 2. Materials and Methods

### 2.1. Chemicals and Reagents

The PARP inhibitor, rucaparib, was kindly gifted from Pfizer Global R&D and the ATR inhibitor, VE-821, was generously supplied by Merck (Merck KGaA, Darmstadt, Germany). Olaparib, niraparib, talazoparib and pamiparib were purchased from Selleckchem (Houston, TX, USA). Drugs dissolved in dry DMSO were stored at −80 °C. Routine chemicals were of the highest purity from Sigma-Aldrich (Poole, UK), unless otherwise stated.

### 2.2. Cell Culture

IGROV-1 and ES-2 cells were obtained from the American Type Culture Collection (ATCC; Manassas, VA, USA) and used within 30 passages of purchase or subsequent authentication by STR profiling (LGC Standards). They were maintained in the exponential phase in RPMI-1640 medium (Merck, Kenilworth, NJ, USA) and 10% foetal bovine serum (FBS; Gibco, Life Technologies, CA, USA) at 37 °C, 5% CO_2_ and 95% humidity. Cells were mycoplasma-free.

### 2.3. PARP Activity Assay

A GCLP-validated assay was used to measure DNA damage-activated PARP activity in permeabilised cells in the presence of an NAD+ substrate (350 nM) and a 12 mer palindromic double-stranded oligonucleotide (10 µg/mL) (Invitrogen, Waltham, MA, USA) to activate PARP1 via immunological detection of the product (PAR) using 10H Ab (Enzo life sciences, Farmingdale, NY, USA) and secondary HRP-conjugated goat anti-mouse Ab (Dako, Santa Clara, CA, USA), as described previously [18,24]. IGROV-1 cells were exposed to 1 µM rucaparib, olaparib, niraparib, pamiparib or talazoparib and ES-2 cells to rucaparib, olaparib or niraparib for 1 h before the drug was washed off with PBS and replaced with fresh media prior to cells being harvested immediately or after 1, 24, 48 or 72 h of incubation in a drug-free medium. Cells were permeabilised and PARP activity measured in comparison to the untreated cells, and cells with 1 μM drug were added directly to the reaction mixture. The percentage of PARP activity was calculated relative to untreated cells.

### 2.4. Western Blot Assay

Exponentially growing IGROV-1 cells were exposed to media containing DMSO or 1 µM of rucaparib, olaparib, niraparib, pamiparib or talazoparib for 1 h, prior to removal and replacement with drug-free media for 0, 24, 48 and 72 h and before extraction with 250 µL/dish of RIPA buffer with a protease cocktail inhibitor (1:100, Thermo Fisher Scientific, Waltham, MA, USA) at 4 °C for 5–10 min. Protein content was measured with a BCA protein assay kit (Thermo Fisher Scientific) and samples were diluted with diH_2_O to 0.5–1.0 mg/mL. A 25% XT sample buffer and 0.5% XT reducing agent (both Bio-Rad, Hercules, CA, USA) were added and samples were heated at 90 °C for 5 min. An amount of 30 µg was loaded/well with 3–8% Criterion XT gels (Bio-Rad) alongside 5 µL/well of HiMark pre-stained protein standard, and the gel was run for 1 h at 170 V (Thermo Fisher Scientific). Separated proteins were transferred to a nitrocellulose membrane (Amersham, Buckinghamshire, UK) for 1 h at 100 V. The membrane was blocked for 1 h in 5% milk powder in TBS-Tween (TBST). Primary antibodies PARP-1 (1:500, sc-53643, Santa Cruz, TX, USA) and GAPDH (1:1000, 14C10, Cell Signaling Technology, Danvers, MA, USA) were diluted accordingly in 5% BSA in TBST overnight at 4 °C on a shaking platform. After washes in TBST the following day, anti-rabbit goat polyclonal HRP and anti-mouse polyclonal HRP were diluted 1:2000 in 5% milk in TBST and added to the membrane sections for 1 h at room temperature. Afterwards, washes with TBST Clarity Max ECL Western substrate were applied to the membrane for 5 min (Bio-Rad) before chemiluminescence was measured using the ChemiDoc Imager (Bio-Rad) or the GBox (Syngene, Cambridge, UK). Densitometry was determined using Fiji ImageJ software and data were plotted using GraphPad Prism 9.0.

### 2.5. Cytotoxicity Scheduling Assays

Exponentially growing IGROV-1 and ES-2 cells were seeded at various densities estimated to give 20–200 colonies following drug treatment. Cells were exposed to 0.5% DMSO (control), VE-821 (1, 3 and 10 µM) or a PARPi (1 µM) single agent in 0.5% DMSO for 24 h. Co-exposed cells were treated with VE-821 (1, 3 or 10 µM) and 1 µM PARPi for 24 h, before the drug was removed and replaced with fresh media for colony formation. Sequentially exposed cells were treated with PARPi for 24 h before replacement with media containing VE-821 for another 24 h, then with drug-free medium for colony formation. Delayed sequentially exposed cells were similarly treated with PARPi for 24 h, but media was replaced with fresh drug-free media for a further 24 h prior to the 24 h exposure to VE-821, and then with the drug-free medium for colony formation media. Colonies were fixed after 10–14 days in methanol: acetic acid (3:1) and stained with 0.4% crystal violet before colonies of >30 cells were counted by eye. Cell survival was calculated from the number of colonies relative to the number of cells seeded. Data were normalised to the DMSO control or PARPi alone, as appropriate, and plotted using GraphPad Prism 9.0 software (San Diego, CA, USA). The potentiation factor, PF_50_, is a unitless variable calculated as the lethal concentration of 50% (LC_50_) for VE-821 alone/LC_50_ VE-821 + PARPi. AUC values and statistical analyses were calculated using GraphPad Prism 9.0.

## 3. Results

### 3.1. Durability of PARP Inhibition by Rucaparib, Olaparib, Niraparib, Pamiparib and Talazoparib

PARPi are currently used most frequently in the treatment of high-grade ovarian cancer; therefore, ovarian cancer cell lines were selected for this study. We first investigated whether our previously observed persistent PARP inhibition by rucaparib was unique or a class effect by comparing the durability of PARP inhibition by rucaparib, olaparib, niraparib, pamiparib and talazoparib in ovarian IGROV-1 and ES-2 cells. 

**Figure 2:**
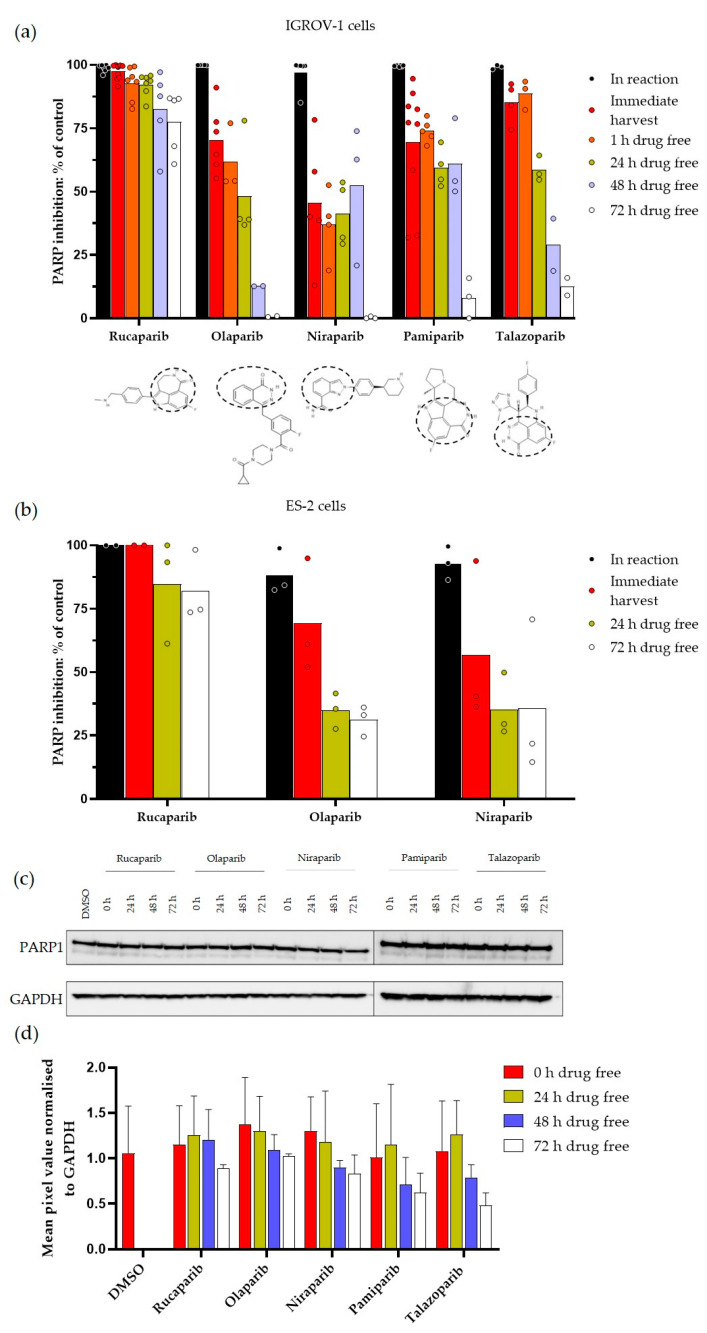
Durability of PARP activity inhibition by PARP inhibitors in IGROV-1 cells and ES-2 cells. PARP activity was measured in cells treated for 1 h with PARPi and harvested immediately or following 1, 24, 48 or 72 h in IGROV-1 cells (**a**) and 24 or 72 h in ES-2 cells (**b**). Percent inhibition was calculated by comparison with untreated cells. Each data point on the graph represents an individual experiment and bars represent the mean of these experiments. The chemical structure of PARPi are given below the X-axis. PARP1 protein levels were measured following exposure to PARPi and subsequent incubation in drug-free medium. (**c**) Representative Western blot of PARP1 levels in IGROV-1 cells treated with the PARPi at specified time points. (**d**) PARP1 levels normalised to GAPDH loading control, data are mean ± SEM of three independent experiments. The original WB can be found in Figure S1.

All of the PARPi inhibited PARP activity completely at 1 µM when added directly into the reaction mix in IGROV-1 cells, and from 89.0 ± 9.0% to 100% in ES-2 cells (Figure 2a,b). However, this level of PARP inhibition in the cells harvested immediately after a 1 h exposure to PARPi was only observed with rucaparib, whereas the other inhibitors only caused reduced levels of inhibition ranging from ~50% (niraparib) to 85% (talazoparib). This could be due to the failure to achieve sufficient intracellular concentration or a wash-out during the harvesting process, as similar levels of inhibition were noted after a further 1 h in the drug-free medium in IGROV-1 cells. The extent of PARP inhibition decreased over time with all inhibitors, but the rate of decrease differed substantially between the inhibitors. In IGROV-1 cells, the decline in PARP inhibition was slowest with rucaparib with an inhibition >90% for 24 h, and fastest with olaparib and niraparib. Notably, after 72 h in the drug-free medium, rucaparib-treated IGROV-1 cells still retained 75% PARP inhibition, whereas there was only a 12% inhibition for pamiparib and talazoparib, and undetectable inhibition after exposure to olaparib and niraparib. Similarly, in ES-2 cells PARP was still inhibited by >80% following 72 h in the drug-free medium, compared to only 30–35% with olaparib and niraparib. The level of PARP inhibition sustained was significantly lower than rucaparib for all inhibitors at all time points and in both cell lines (*p* < 0.0001 to *p* = 0.04), with the exception of talazoparib at 1 h postexposure and niraparib and pamiparib at 48 h post exposure in IGROV-1 cells.

The durability of inhibition was not due to the degradation of the PARP1 protein induced by the inhibitors and followed by the gradual resynthesis of the PARP1 enzyme (Figure 2c). In fact, there seemed to be less PARP1 at later time points, which was more pronounced with pamiparib and talazoparib and may represent a loss of the full-length PARP1 protein due to apoptotic caspase-mediated cleavage (Figure 2d).

### 3.2. Investigating Schedules of PARPi and ATRi

**Figure 3:**
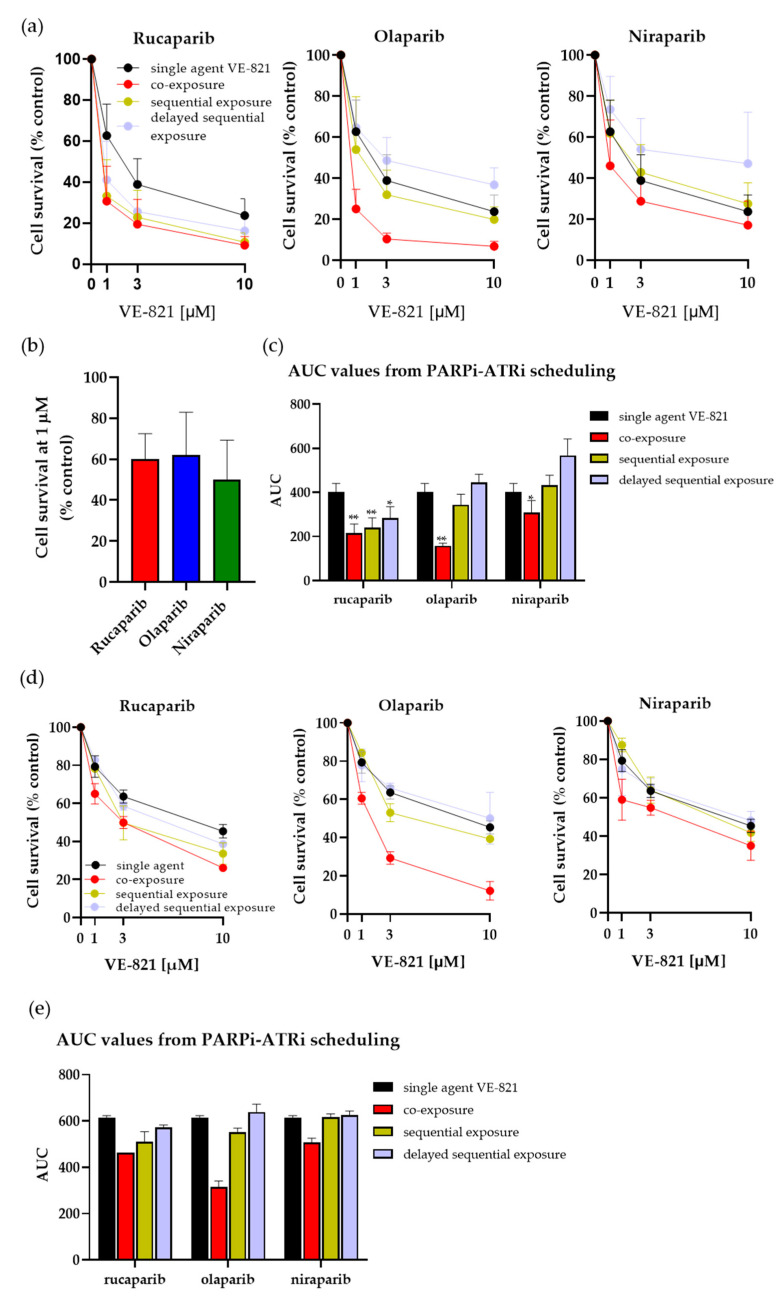
Enhancement of VE-821 cytotoxicity by rucaparib, olaparib and niraparib on different schedules. Cells were exposed to VE-821 at indicated concentrations, either as single agents or with 1 µM rucaparib, olaparib or niraparib, and either with co-exposure, sequential exposure or 24 h delayed sequential exposure; then, they were incubated for 10–14 days for colony formation in IGROV-1 (**a**) and ES-2 (**d**) cells. (**b**) IGROV-1 cells were exposed to 1 µM rucaparib, olaparib and niraparib as single agents for 24 h, then drug-free medium cells for 10–14 days, and cell survival was calculated by reference to vehicle-alone (DMSO) controls. The cytotoxicity of combinations with VE-821 was normalised to PARPi alone and the cytotoxicity of VE-821 alone was normalised to DMSO control. Data are mean ± S.D of five independent experiments with IGROV-1 cells and two independent experiments in ES-2 cells. Comparative AUC values were calculated from IGROV-1 (**c**) and ES-2 (**e**) cells treated with co-exposure, sequential exposure or 24 h delayed sequential exposure. * *p* < 0.05, ** *p* < 0.01. Statistical analyses were performed using GraphPad Prism 9.0.

To determine if the difference in the durability of target inhibition by the PARPi would affect the cytotoxicity in combination with ATRi VE-821 on different schedules, we compared the effect of the PARPi on VE-821 cytotoxicity using three different schedules: concurrent PARPi and VE-821, sequential PARPi followed by VE-821, and sequential PARPi with a 24 h drug-free gap between PARPi and VE-821 exposure. Rucaparib, the most durable, and olaparib and niraparib, the least durable, were selected for this investigation in IGROV-1 and ES-2 cells (Figure 3). VE-821 was one of the first potent ATRi and is from the same chemical series as clinical ATRi berzosertib.

The cytotoxicity of combinations with VE-821 was normalised to PARPi alone and the cytotoxicity of VE-821 alone was normalised to the DMSO control. Rucaparib, olaparib and niraparib all sensitised IGROV-1 and ES-2 cells to VE-821 in the co-exposure setting (Figure 3a,d). All PARPi increased the cytotoxicity of VE-821 when both drugs were co-exposed with olaparib, causing the greatest sensation, and niraparib, the least sensitisation, in both cell lines (PF_50_ values: rucaparib = 2.3 to 2.7, olaparib = 2.8 to 4.7 and niraparib = 1.5 to 1.8) (Figure 3a,c–e). This was not due to any difference in the intrinsic cytotoxicity of the PARPi alone as there was no significant difference between the inhibitors in terms of cytotoxicity, with 1 µM causing between 40% and 50% of the inhibition of cell viability (Figure 3b).

With sequential exposure, where cells were incubated with the PARPi alone for 24 h and immediately followed by 24 h exposure to VE-821 alone, only rucaparib caused significant sensitisation and was equally as effective as when co-exposed with VE-821 in both IGROV-1 and ES-2 cells (PF_50_: 2.6 and 2.3, respectively), (Figure 3a,d). In marked contrast, neither niraparib nor olaparib significantly increased VE-821 cytotoxicity when given sequentially. 

When IGROV-1 and ES-2 cells were incubated for 24 h in the drug-free medium between the PARPi and VE-821 exposures, rucaparib still caused sensitisation at the LC_50_ and at 10 µM VE-821 in IGROV-1 cells (2.2 and 1.8-fold), indicating sufficient PARP inhibition is sustained over the 24–48 h period after rucaparib withdrawal. Less sensitisation was observed at LC_50_ and at 10 µM VE-821 in ES-2 cells (1.6- and 1.2-fold). No potentiation was observed with olaparib or niraparib; in fact, there seemed to be some modest protection (Figure 3a,d).

Analysis of the AUC of the cytotoxicity curves revealed that, whereas rucaparib, olaparib and niraparib significantly sensitised IGROV-1 cells to VE-821 in a co-exposure setting (*p* = 0.001, *p* = 0.004 and *p* = 0.04, respectively), only rucaparib caused significant sensitisation in sequential and delayed sequential settings (*p* = 0.002 and *p* = 0.02, respectively) (Figure 3c). Similarly, in ES-2 cells there was a reduction in the AUC values in all settings with the addition of rucaparib compared to single-agent VE-821, which was only observed with olaparib or niraparib when co-exposed to VE-821 (Figure 3e). These data may indicate olaparib and niraparib exposure had arrested the cells such that they did not progress through the S phase during the incubation with VE-821.

## 4. Discussion

Following previous studies highlighting the durability of PARP inhibition by rucaparib [18,19], we investigated if this was a unique property of this PARPi or was common to other clinically approved PARPi. Our results reveal that although all PARPi continued to inhibit cellular PARP activity to a certain extent after drug removal, the sustained high level of inhibition of PARP activity over a period of 3 days was unique to rucaparib. There was a progressive loss of PARP inhibition between 24 and 72 h post-drug removal with all inhibitors, which was most rapid for olaparib, followed by niraparib, and was intermediate for talazoparib and pamiparib. Only for rucaparib was inhibition maintained at ≥75% for 72 h in both IGROV-1 and ES-2 cells. Pamiparib resembles rucaparib structurally in that the carboxamide group of the nicotinamide pharmacophore is incorporated into a seven-membered ring (Figure 2a). However, it did not cause the sustained level of PARP inhibition that we observed with rucaparib, indicating that this structural element was not responsible for the durability of inhibition. PARP1 has a long half-life of around 60 h [25], which could be consistent with the data if the inhibitors were causing its degradation, as has been observed with other proteins treated with their inhibitors [26]. Indeed, the development of drugs specifically designed to cause target protein degradation using proteolysis-targeting chimera or PROTAC is an exciting area of drug development [27], and a specifically designed PARP PROTAC, the iRucaparib-AP6 compound based on rucaparib, has been developed recently [28]. However, our investigation of protein levels revealed that the durable inhibition after the removal of the drug was not due to PARP1 protein degradation by any of the inhibitors (Figure 2c). These results also suggest durability is not related to trapping potency, as talazoparib is reported to be the most potent trapper, but clearly not the most durable [2].

For single-agent activity, PARP must be inhibited continuously, and all PARPi are approved for daily or twice-daily administration. The approved doses and schedules of the PARPi are established in early phase trials based on PK and tolerability rather than optimum pharmacodynamic effect. Rucaparib is recommended at a dose of 600 mg twice daily, but its unique ability to cause sustained PARP inhibition suggests that twice-daily dosing may not be necessary and an intermittent dosing schedule, perhaps twice weekly, might be equally effective, as well as more tolerable and affordable. Indeed, our earlier xenograft studies would suggest that this is plausible [19]. Introducing a more tolerable schedule could also be beneficial in reducing the risk of haematological disorders arising from long-term PARPi use [29]. Further clinical evaluation of intermittent scheduling of PARPi is warranted. The PARP inhibitory effects of talazoparib are also durable, causing >80% PARP inhibition for 24 h in IGROV-1 cells, and could potentially be given on alternate days. A reduced dose-intense schedule of PARPi would be particularly beneficial in low- to middle-income countries where, for the majority of patients, PARPi at the current recommended doses are unaffordable. 

Following sound preclinical evidence that demonstrates the synergy between PARPi and ATRi inhibitors [30,31,32,33], three PARPi are currently being investigated clinically in combination with ATR inhibitors. Rucaparib is not currently being investigated in combination with ATR inhibitors in the clinical setting. However, olaparib is in various phase 2 trials with the ATR inhibitor, AZD6738, for a variety of cancer types (NCT03682289, NCT03787680, NCT03462342, NCT04065269, NCT0387095, NCT04498021, NCT04090567, NCT02576444, NCT04417062, NCT03740893, NCT03579316, NCT03330847 and NCT02937818) and niraparib is in phase 1 trials in combination with various ATR inhibitors (NCT03682289, NCT03787680, NCT03462342, NCT04065269, NCT04170153, NCT04149145 and NCT04267939). Our investigation of various schedules of administration of PARPi with the ATR inhibitor VE-821 revealed that olaparib and niraparib only synergised with VE-821 when cells were exposed to both drugs concurrently. In contrast, rucaparib was similarly effective in enhancing VE-821-mediated cytotoxicity when given simultaneously or immediately prior to VE-821 exposure, and even increased the cytotoxicity of VE-821 when there was a 24 h delay in adding the VE-821. On the basis of these data, it may not be appropriate to extrapolate from trials with olaparib and niraparib when designing rucaparib combination trials. Furthermore, it may be beneficial to consider schedules where rucaparib is given on an intermittent basis (every 2 or 3 days) in combination with an ATRi. Such schedules may be equally effective but less toxic. However, further investigations are required to determine whether sequential dosing would have equivalent anticancer activity in vivo and to examine the implications for toxicity prior to exploring different schedules clinically. 

The toxicities associated with PARPi in combination with cytotoxic chemo- and radiotherapy clinically have resulted in a failure of PARPi to progress beyond phase 2 clinical evaluation in combination. Preclinical data indicate that for a synergy with cytotoxic chemo- and radiotherapy, lower doses and shorter durations of PARP inhibition, i.e., during the repair phase of the induced damage, are all that are necessary, and that higher doses are highly toxic [34,35]. The data reported here have implications for the investigation of such combinations clinically. It is possible that the persistence in PARP inhibition accounts for some of the clinical toxicities observed and that intermittent PARPi schedules would be preferable.

## 5. Conclusions

In conclusion, we report here that five clinically active PARPi continue to inhibit cellular PARP activity after the drug has been removed and that this inhibition diminishes with time to a variable degree. Suppression of PARP activity is most durable with rucaparib and least durable with olaparib and niraparib. This sustained PARP inhibition was unique to rucaparib and not a class effect amongst approved PARPi. This durable suppression meant that rucaparib was effective at enhancing ATR inhibitor-induced cytotoxicity when given prior, including with a 24 h delay, with the ATR inhibitor. In contrast, olaparib and niraparib only increased ATR inhibitor-induced cytotoxicity when given simultaneously. These data have implications for the scheduling of PARPi alone and in combination with ATR inhibitors and cytotoxic drugs.

## Data Availability

The data presented in this study are available on request from the corresponding author.

## Supplementary Materials

The following are available online at https://www.mdpi.com/article/10.3390/cancers14225559/s1, Figure S1: The original Western Bolt of Figure 2.
