# Supplementary material for: Differences in Durability of PARP Inhibition by Clinically Approved PARP Inhibitors: Implications for Combinations and Scheduling
Source: Cancers (Basel). 2022 Nov 12;14(22):5559. doi: 10.3390/cancers14225559 (PMC9688250; doi:10.3390/cancers14225559)

Figure S1 Representative original Western blots (Figure 2c)

PARP gel 1

loading order: DMSO, 0 h rucaparib, 24 h rucaparib, 48 h rucaparib, 72 h rucaparib, 0 h olaparib, 24 h olaparib, 48 h olaparib, 72 h olaparib, 0 h niraparib, 24 h niraparib, 48 h niraparib, 72 h niraparib.

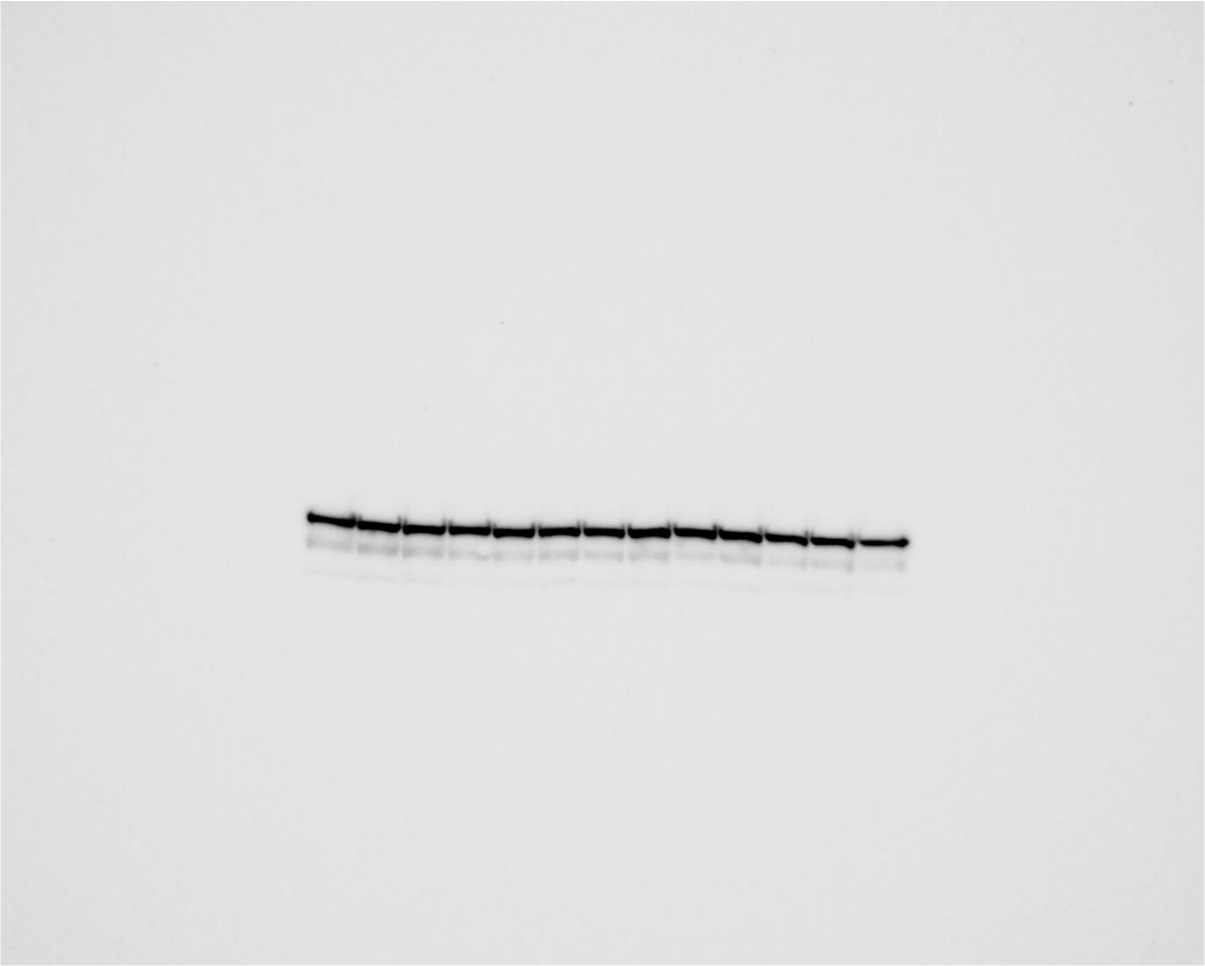

GAPDH gel 1:

loading order: DMSO, 0 h rucaparib, 24 h rucaparib, 48 h rucaparib, 72 h rucaparib, 0 h olaparib, 24 h olaparib, 48 h olaparib, 72 h olaparib, 0 h niraparib, 24 h niraparib, 48 h niraparib, 72 h niraparib.

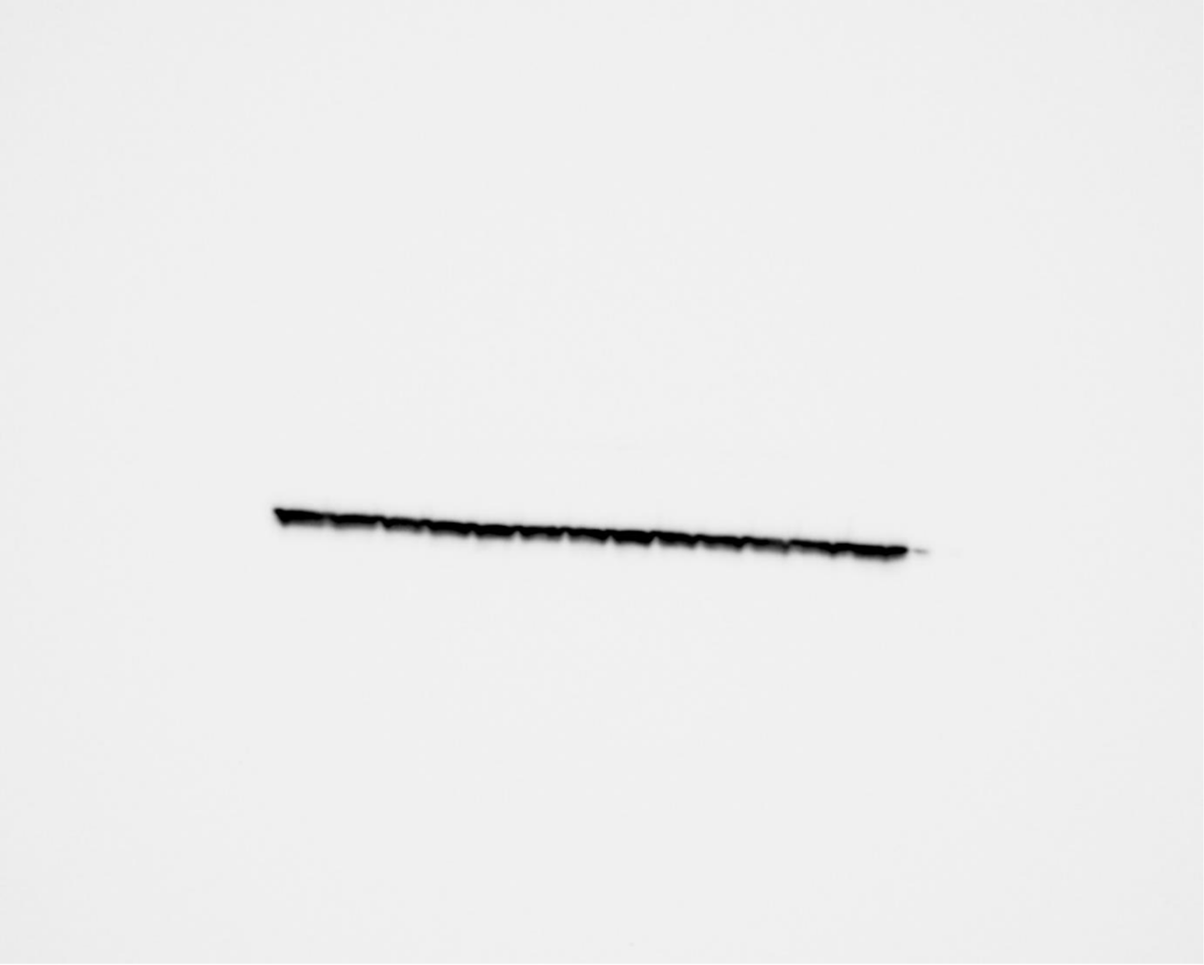

PARP gel 2:

Loading order: 0 h pamiparib, 24 h pamiparib, 48 h pamiparib, 72 h pamiparib, 0 h talazoparib, 24 h talazoparib, 48 h talazoparib and 72 h talazoparib

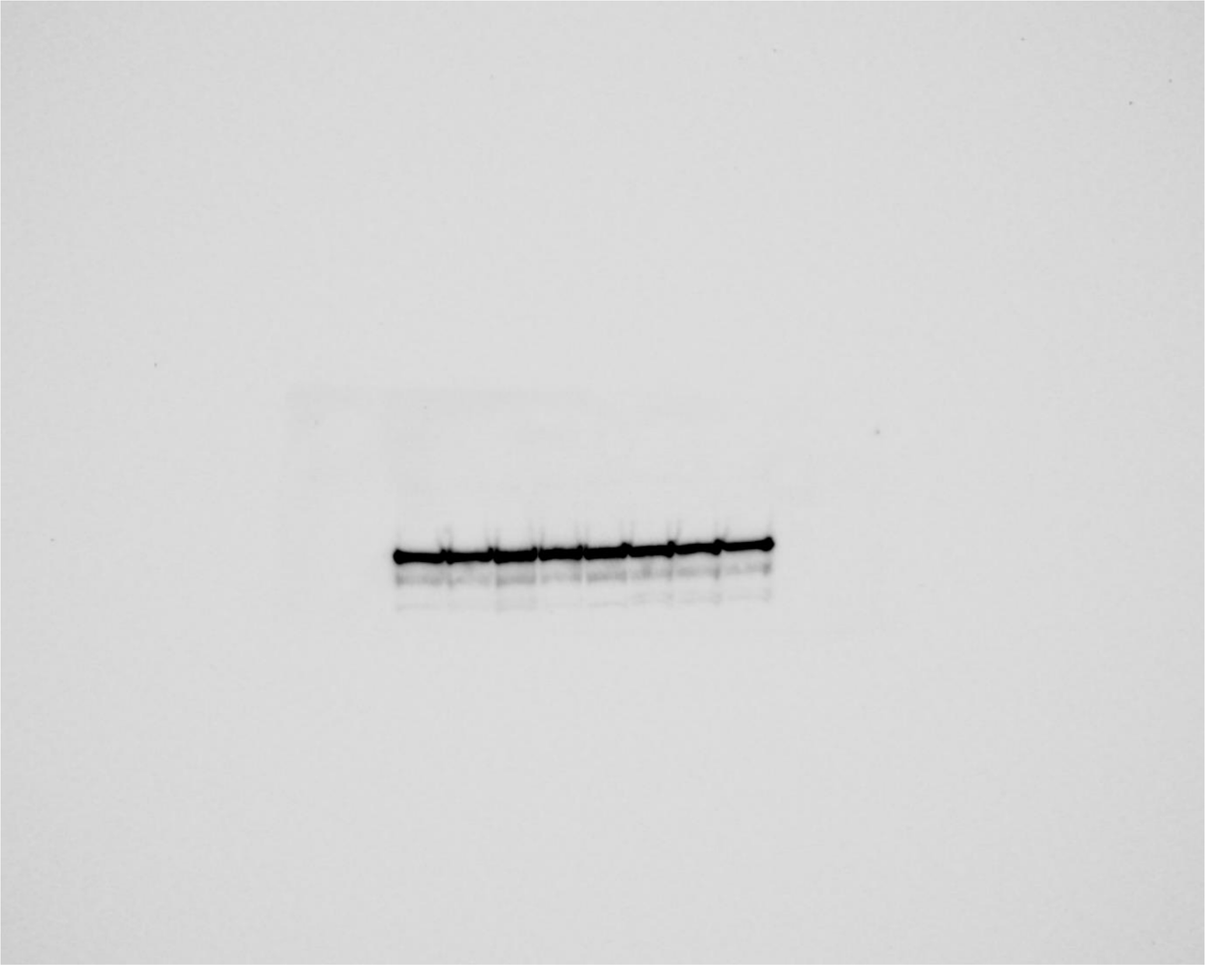

GAPDH gel 2:

Loading order: 0 h pamiparib, 24 h pamiparib, 48 h pamiparib, 72 h pamiparib, 0 h talazoparib, 24 h talazoparib, 48 h talazoparib and 72 h talazoparib

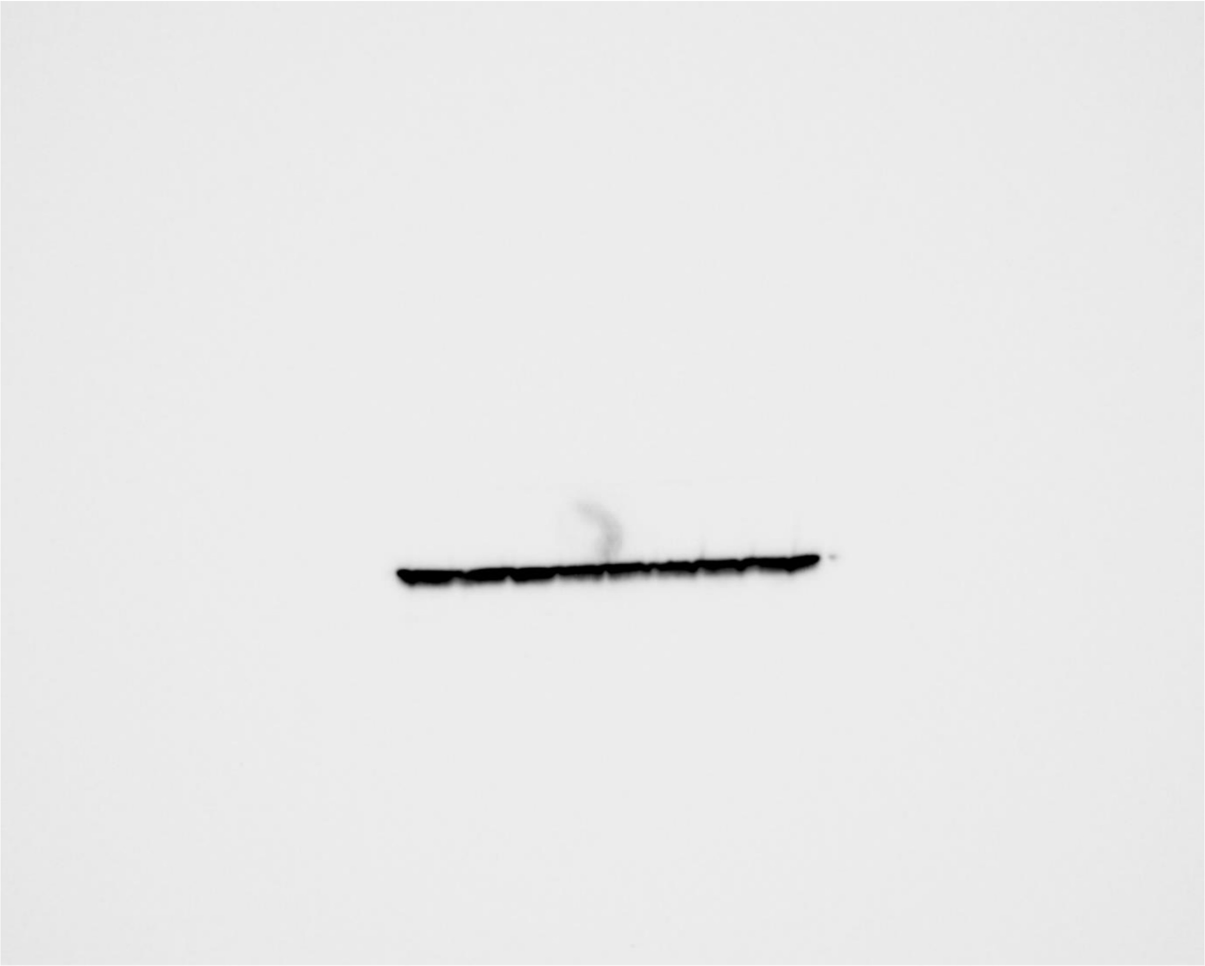

Supplement: Supplementary file 1 [file cancers-14-05559-s001.zip › cancers-1973576-supplementary.pdf]
